# Repetitive transcranial direct current stimulation modulates the brain–gut–microbiome axis in obese rodents

**DOI:** 10.1007/s43440-022-00401-z

**Published:** 2022-08-09

**Authors:** Agata Ziomber-Lisiak, Katarzyna Talaga-Ćwiertnia, Agnieszka Sroka-Oleksiak, Artur D. Surówka, Kajetan Juszczak, Magdalena Szczerbowska-Boruchowska

**Affiliations:** 1 Chair of Pathophysiology, Faculty of Medicine, Jagiellonian University Medical College, Kraków, Poland; 2 Chair of Microbiology, Faculty of Medicine, Jagiellonian University Medical College, Kraków, Poland; 3 Faculty of Physics and Applied Computer Science, AGH University of Science and Technology, Kraków, Poland; 4 Department of Urology and Andrology, Collegium Medicum, Nicolaus Copernicus University, Bydgoszcz, Poland

**Keywords:** tDCS, Feeding behavior, High-calorie diet, Obesity, Brain–gut–microbiome axis

## Abstract

**Background:**

Complex interactions between the brain, gut and adipose tissue allow to recognize obesity as a neurometabolic disorder. The recent data have shown that gut microbiota can play a potential role in obesity development. Transcranial direct current stimulation (tDCS) is a safe and non-invasive technique to modulate the activity of cerebral cortex and other connected brain areas also in context of appetite control. The objective of this study was to evaluate the effects of repetitive anodal tDCS (AtDCS) of prefrontal cortex on feeding behavior, metabolic status and selected phyla of gut microbiota in rats with obesity induced by high-calorie diet (HCD).

**Methods:**

32 female Wistar rats were equally divided into 4 subgroups depending on diet effect (lean versus obese) and type of stimulation (active versus sham tDCS versus no stimulation). Feed intake, body weight, blood lipoproteins and leptin levels as well as Firmicutes and Bacteroidetes in intestines and stool were examined.

**Results:**

HCD changed feeding behavior and metabolic parameters typically for obesity-related ranges and resulted in an abundance of Firmicutes at the expanse of Bacteroidetes in the large intestine and stool. AtDCS decreased appetite, body weight, and cholesterol levels. In addition, AtDCS reduced ratio of the average number of Firmicutes to average number of Bacteroidetes in all examined tissues.

**Conclusions:**

Repetitive AtDCS is not only effective for appetite restriction but can also modulate gut microbiome composition which demonstrates the existence of the brain–gut–microbiome axis and points at this technique as a promising complementary treatment for obesity. However, the effects should be further replicated in human studies.

## Introduction

Obesity is reaching epidemic rate worldwide. It is a complex and multifactorial disease associated with increased morbidity and mortality. Unhealthy life style (i.e., high-fat diet and lack of physical activity), neuronal and hormonal abnormalities, as well as genetic and epigenetic factors are involved in obesity development [1]. Obesity is nowadays recognized as a brain disease, rather than a simple metabolic disorder due to documented multiple interactions between the brain, gut and adipose tissue. Obesity is characterized by a chronic low-grade inflammation and a rodent study revealed [2] that the hypothalamus is injured in obese subjects fed a high-fat diet. It was shown that obesity is linked not only to cognitive decline but also other brain disorders such as dementia, anxiety, and depression [3, 4]. It is assumed that brain structural changes observed in obesity result from the synergistic interplay between the different obesity-induced risk factors such as oxidative stress, inflammation, insulin resistance and lipotoxicity [5, 6]. An experimental study suggests a high-fat diet could induce symptoms of depression, such as anxiety and anhedonia, which may be linked to reduced dopaminergic response [7].

Appetite is under regulation of homeostatic, reward, and cognitive circuits. Lateral and medial hypothalamus (hunger and satiety centers) with arcuate and paraventricular nuclei are key regions responsible for homeostatic control. Reward-related limbic pathways including the ventral and dorsal striatum, midbrain, amygdala, and orbitofrontal cortex may override normal satiety signals resulting in hyperphagia and excessive body weight [8]. Cognitive control of feeding behavior is linked to learned eating habits, choices to cultural beliefs and other environmental influences [9].

Leptin is considered as a key regulator of the brain–gut axis. White adipose tissue (WAT) is the main source of leptin, but this hormone has been shown to be also produced in human and rodent brain exerting multiple metabolic effects [10, 11]. Leptin positively correlates with body mass index (BMI) [12], affects body weight homeostasis [13, 14], numerous inflammatory and immune processes [15] as well as reproductive functions [16]. Activation of hypothalamic leptin receptors suppresses food intake and promotes energy expenditure pathways [13, 14], however most human obesity is characterized by hyperleptinemia and resistance to leptin action [16, 17]. Dyslipidemia is very common in obesity and a feature of metabolic syndrome. High levels of serum triglycerides, free fatty acids, very low-density lipoproteins (VLDL), apolipoprotein B (Apo B), and non-high-density lipoprotein (non-HDL) cholesterol are typical obesity-related lipid abnormalities [18, 19]. Hyperleptinemia, hypoadiponectinemia, and insulin resistance are also widely linked to features of the metabolic syndrome [20].

It has been shown that changes in satiation are linked to diet composition and that fat generates subcaloric compensation and hence promotes excess energy intake [21]. A high-fat diet promotes the development of obesity and there is a direct relationship between the amount of dietary fat and the degree of obesity. Dietary fat induces overconsumption and weight gain through its low satiety properties and high caloric density [22]. Recent data have revealed the relationship between diet, gut microbiota and energy homeostasis [23, 24] showing that obesity is associated with alterations in the composition and functional properties of the gut microbiota. The human gut microbiome is mostly composed of two dominant bacterial phyla, Firmicutes and Bacteroidetes, which represent more than 90% of the total bacterial community [25]. The normal gut microbiome is responsible for the overall health of the host playing a crucial role in nutrient and drug metabolism, immunomodulation and preventing pathogen colonization [25–27]. Dysbiosis (chronically modified gut microbiome composition) is observed in various pathologic conditions of the gastrointestinal tract, immune system, central nervous system or metabolic disorders such as obesity, type 2 diabetes or atherosclerosis [28–32]. Clinical and experimental data have shown that fecal microbiota of both obese human and animals exhibit an increased Firmicutes and decreased Bacteroidetes phyla and therefore increased Firmicutes to Bacteroidetes ratio compared with normal-weight individuals [23, 27, 30, 32, 33]. This ratio is commonly proposed in scientific literature as a potential biomarker of obesity [31, 32, 34]. Additionally, it was reported that diet contents rather than adiposity affect the microbiota composition [33, 35]. De Filippo et al. have demonstrated fewer bacteria of the Enterobacterales, a higher proportion of Bacteroidetes and a smaller proportion of Firmicutes in gut microbiota profile of children on vegetarian diet compared with those on so-called Western diet [36].

Current evidence demonstrated that the alterations of brain electric activity lie behind many neuropsychiatric disorders [37]. One theory implies that obesity is a consequence of interhemispheric activity imbalance [38]⁠ and reduced function of the right prefrontal cortex (PFC) may lead to overeating and inactivity, whereas an excess activation of this brain region can reduce eating drive and promote mobilization. Neuroimaging studies have shown that people effectively maintaining long-term weight loss show a pattern of increased activation in the lateral PFC during satiation or in response to food cues [39, 40]. This activation is stronger than in control (non-obese) subjects, suggesting that lateral prefrontal hyperactivity may be a compensatory mechanism to overcome obesity in these individuals. Therefore, target modulation of cortical excitability or neuroplasticity with external stimuli could mimic this compensatory mechanism, and hence provide a novel therapeutic option of obesity [40]. Transcranial direct current stimulation (tDCS) has received great attention as a novel and safe alternative for treatment of major depression, chronic pain, stroke, and neurodegenerative disorders [41–44]. By triggering the current flow, tDCS modifies neuronal activity according to the modality of the application. Anodal-type tDCS (AtDCS) is assumed to increased excitability and spontaneous firing of the cortical neurons, while the cathodal-type tDCS (CtDCS) induces opposite effects [45]. Translating tDCS into the field of appetite control can be particularly important due to dramatic increase in obesity prevalence. The existing research provided promising data showing a significant reduction in food craving following just one session of the dorsolateral frontal cortex (DLFC) tDCS [45, 46].

The objective of this study was to evaluate feeding behaviors, metabolic parameters and selected phyla of gut microbiota in rats maintained on high-calorie diet subjected to repetitive AtDCS of the right PFC. AtDCS of the right PFC was shown to restrict food cravings in both animal [47] and human studies [48], therefore, we expected that AtDCS-exposed animals may show decreased appetite, improvement in obesity-related metabolic disturbances and alternations in gut microbiome composition.

## Materials and methods

### Animal housing

The study included 32 female 12–13-week-old Wistar rats with mean baseline body weight of 231 ± 4 g. The rats were kept in plastic cages (two in each cage) in an air-conditioned room with 21–25 °C temperature, 55–65% humidity and 12-h to 12-h light to dark cycle. The rats were randomized to feeding with standard chow pellets (*N* = 8) (fats 8%, carbohydrates with ash and minerals 67%, proteins 25%; energy 2.75 kcal/g; Labofeed B, Kcynia, Poland) or high-calorie diet (HCD) (*N* = 24) with nearly three-fold higher content of fats than the standard feed (fats 22%, carbohydrates with ash and minerals 46%, proteins 32%; energy 4.7 kcal/g; Perform, Opti Life, Kronen, Belgium). During the course of the study (6 weeks), animals from both groups had unlimited access to feed and drinking water [49]⁠.

The experiments were conducted in accordance with the National Guide for the Care and Use of Laboratory Animals, and were approved by the Local Ethical Committee on Animal Testing at the Jagiellonian University in Krakow, Poland (approval no. 157/2013). All possible attempts have been made to minimize animal suffering and discomfort, and the number of examined rats was reduced to the necessary minimum according to the 3R principles.

### tDCS technique

**Fig. 1:**
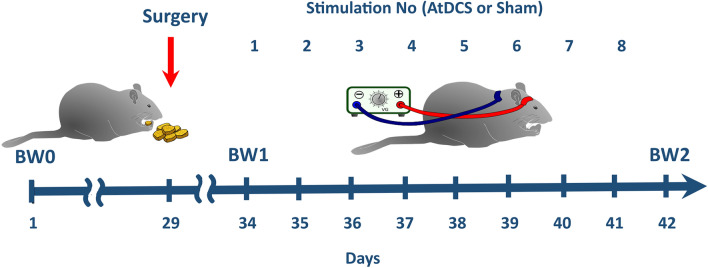
Study design. The rats were fed standard or high-calorie diet (HCD) during the whole experiment (42 days). 5 days before the first stimulation (29th day), the implantation of epicranial electrode on the prefrontal area was performed in Sh and St. The active stimulation started on 34th day of the study and continued through 8 consecutive days till 41st day (two sessions of a-10 min stimulation daily with current intensity of 200 µA). The body weight (BW, g) and feed intake (FI, kcal) were measured 24 h after each consecutive stimulation, therefore the first measurement took place on 35th day, while the last one—on 42nd day of the study (BW2, FI2). BW0 indicates initial body weight at the beginning of the study, while BW1—body weight measured just before the first stimulation (on 34th day of the study)

tDCS was carried out with a constant current stimulator (BrainStim, EMS, Bologna, Italy) for continuous application of low currents. Both surgical and stimulation procedures were described in details in our previous study [50]. An illustration of the experiment design is presented in Fig. 1. In general, the currents were delivered transcranially via an epicranial electrode fixed on the frontal part of the scalp, while reference electrode, a conventional rubber plate, was placed on animal’s back and stabilized with a corset. Prior to the stimulation, the electrodes were filled with 0.9% NaCl and connected to the DC stimulator controlled by a dedicated software. During AtDCS, current was delivered from epicranial to back electrode. The stimulation was conducted in conscious rats throughout 8 consecutive days, during which each animal was subjected to two 10-min daily sessions of active (200 µA) or sham (0 µA) AtDCS (c.f. Fig. 1). During the stimulation period, the rats were placed in separate plastic cages and monitored carefully for any behavioral abnormalities. Our previous studies [50, 51] proved tDCS procedure as safe and not harmful methodology as no abnormalities such as signs of neurotrauma, edema, hematoma or other pathological changes of the brain tissue were detected in histological examinations.

### Study groups and metabolic parameters

On the day of epicranial electrode implantation, the obese rats were randomly divided into three subgroups depending on procedure applied: Ob (*N* = 8)—intact animals on HCD, non-exposed to stimulation; Sh (*N* = 8)—animals on HCD, subjected to sham AtDCS; St (*N* = 8)—animals on HCD, exposed to active AtDCS of the right PFC. Together with the lean intact rats on standard chow diet (L; *N* = 8) four subgroups were created. The following average body weights were recorded in tested groups on the day of the first AtDCS application: Ob—291 ± 4 g, Sh—289 ± 4 g, St—290 ± 6 g and L—276 ± 4 g. Dietary intake and body weight were recorded throughout the course of the study: twice a week before and daily during stimulation period using digital scale. To evaluate the amount of feed consumed in each cage, the caloric intake was evaluated during the experiment and the daily feed intake (dFI g/24 h) was calculated as the difference between the diet offered and the leftovers collected from the cage divided by the number of days between two consecutive measurements. The weight in grams was then converted to kcal and this value was divided by the number of rats in the cage to estimate the intake in kcal per rat per cage. Next, average cumulative feed consumption (cFI, kcal) was assessed by multiplication of dFI (kcal/24 h) by the number of days of the experiment. Average daily body weight gain (dBWG, g/ 24 h) was calculated as the difference in body weights recorded at two consecutive measurements divided by the number of days of the between-measurement period. Specific rate of body weight gain (sBWG; g/kg) was calculated according to anthropometrical parameter of obesity in rats proposed by Novelli et al. [52] as a function of d*M*/*M*d*t*, where dM represents the gain of body mass during d*t* = *t*2—*t*1 (time 2—the last day of the study; time 1—the first day of the study) and *M* is the rat body mass at t1. At the end of the experiment (42 day of the study), 24 h after the last—8th stimulation, all rats were guillotined and samples of blood, both small (jejunum) and large (colon) intestines as well as stool and other tissues were collected under sterile conditions for further examinations.

Metabolic parameters (i.e., body weight, BW or feed intake, FI) were analyzed at the beginning of the experiment and during pre-stimulation and stimulation periods, which was included in the description of these parameters as 0, 1 and 2, respectively (i.e., BW0, BW1 and BW2, respectively; c.f. Fig. 1).

### Biochemical analysis

Serum levels of leptin [pg/mL] were measured using conventional rat ELISA kits (Mouse and Rat Leptin ELISA BioVendor). Blood total cholesterol (TCh) [mmol/L] was assessed with the enzymatic method, triglycerides [mmol/L] GPO PAP method, and HLD [mmol/L] and low-density lipoprotein (LDL) [mmol/L] direct methods on a Roche Cobas c501 analyzer (Roche Diagnostics GmbH, Mannheim, Germany).

### The assessment of selected phyla of gut microbiota

#### Intestine samples and stool

Bacterial DNA was extracted using Genomic Mini (A&A Biotechnology, Gdynia, Poland) for small and large intestine as well as stool samples. Obtained DNA templates were used to quantitative PCR real-time amplification (qPCR) to estimate the number of Firmicutes and Bacteroidetes phyla.

### Quantitative PCR real time

The following specific primers targeting Bacteroidetes as well as Firmicutes the amplification program were used. To determine the Bacteroidetes (5′-AACGCTAGCTACAGGCTT-3′) and (5′-CAATCGGAGTTCTTCGTG-3′) oligonucleotide sequence as described by Dick et al., Firmicutes (5′-GCGTGAGTGAAGAAGT-3′) and (5′-CTACCGCTCCCTTTACAC-3′) was used [53, 54]. 10 µL of reaction mixture was prepared separately for these groups of bacteria: 5 µL SYBR Green PCR Master Mix (A&A Biotechnology); 0.1 µL of specific primers pair (Genomed) at a concentration of 20 mmol and 2 µL of the template. The following PCR conditions were used: initial denaturation at 95 °C for 5 min, followed by 40 cycles consisting of denaturation (95 °C for 30 s), annealing (47 °C for Bacteroidetes and 44 °C for Firmicutes primers; 30 s) and extension (72 °C for 30 s). The PCR amplification was carried out in a CFX96 thermocycler (BioRad) for each sample in duplicate. The bacterial number was calculated by comparing the Cq (quantification cycle) values to the standard curves. DNA from *Bacteroides fragilis* ATCC 25285 for Bacteroidetes and *Enterococcus faecalis* ATCC 29212 for Firmicutes was added in serial dilutions corresponding to 10^1^–10^7^ cells to a series of qPCRs, respectively. To determine the number of *Bacteroides* and *Prevotella* cells, the fluorescent signals detected in DNA samples (in duplicate) in the linear range of the assay were averaged and compared to the standard curves. The obtained data were converted into 1 g of tissue.

### Calculations and statistics

The data distribution (normal or not) was determined by Shapiro–Wilk test. Consequently, all the results were presented as mean (± SD) or median (along with 25th–75th percentiles). In case of normal distribution and equal variances of the data subgroups, the parametric two-way analysis of variance (ANOVA), followed by the Bonferroni post hoc test was used. The data which did not fulfill the criterion of normality or equal variances were analyzed with a nonparametric Kruskal–Wallis test followed by the Bonferroni post hoc test. To compare body weight (BW, g) at different time points repeated-measures analysis of variance (ANOVA) with “TIME” as a within-subject factor and “GROUP” as a between-subject factor followed, when appropriate, by the Bonferroni post hoc test was used. To investigate whether the time effect differed between groups, we confirmed the “TIME” × “GROUP” interaction. The Mauchly test of sphericity was performed and the Greenhouse–Geisser correction was applied when necessary. Pearson’s or Spearman’s correlation was applied to demonstrate the relationship between examined parameters with or without normal data distribution. The correlation coefficients of the sample were classified according to Guilford's interpretation [55]. The data were considered statistically significant when *p* < 0.05. Statistical analyses were conducted using IBM Inc. SPSS Statistics 26.0 software.

## Results

### Feeding behavior and metabolic parameters

**Fig. 2:**
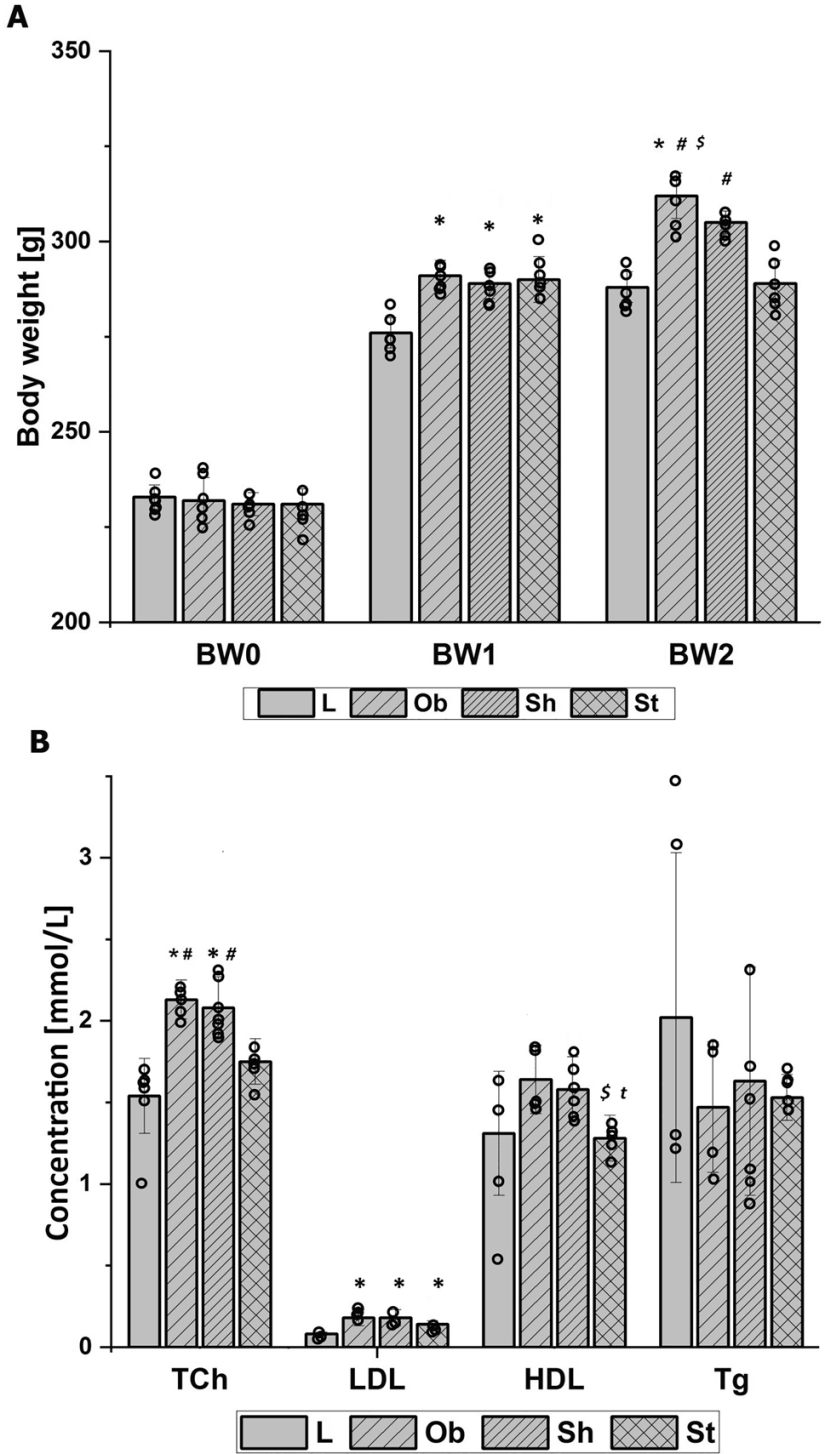
**A** Average body weights [g] within groups at the beginning of the experiment (BW0), before the first stimulation (BW1) and 24 h after the last stimulation (BW2). Two-way ANOVA and repeated-measures ANOVA, followed by the Bonferroni post hoc test. Data are shown as mean ± SD. **◦** raw data; *p = 0.000 vs. L; ^**#**^*p* = 0.000 vs. St; ^$^*p* = 0.044 vs Sh. **B** Concentrations [mmol/L] of serum total cholesterol (TCh), low-density lipoprotein (LDL), high-density lipoprotein (HDL) and triglycerides (Tg) at the end of the study. Two-way ANOVA (TCh) or Kruskal–Wallis test (LDL, HDL, Tg) followed by the Bonferroni post hoc test. Data are shown as mean ± SD. **◦** raw data; *p = 0.000 vs. L; ^**#**^*p* < 0.007 vs. St, ^$^*p* = 0.048 vs Sh, ^t^*p* = 0.067 vs (L + Ob). L—lean intact (*N* = 8); Ob—intact with obesity (*N* = 8); Sh—sham-stimulated with obesity (*N* = 8); St—obese with active stimulation (*N* = 8)

**Fig. 3:**
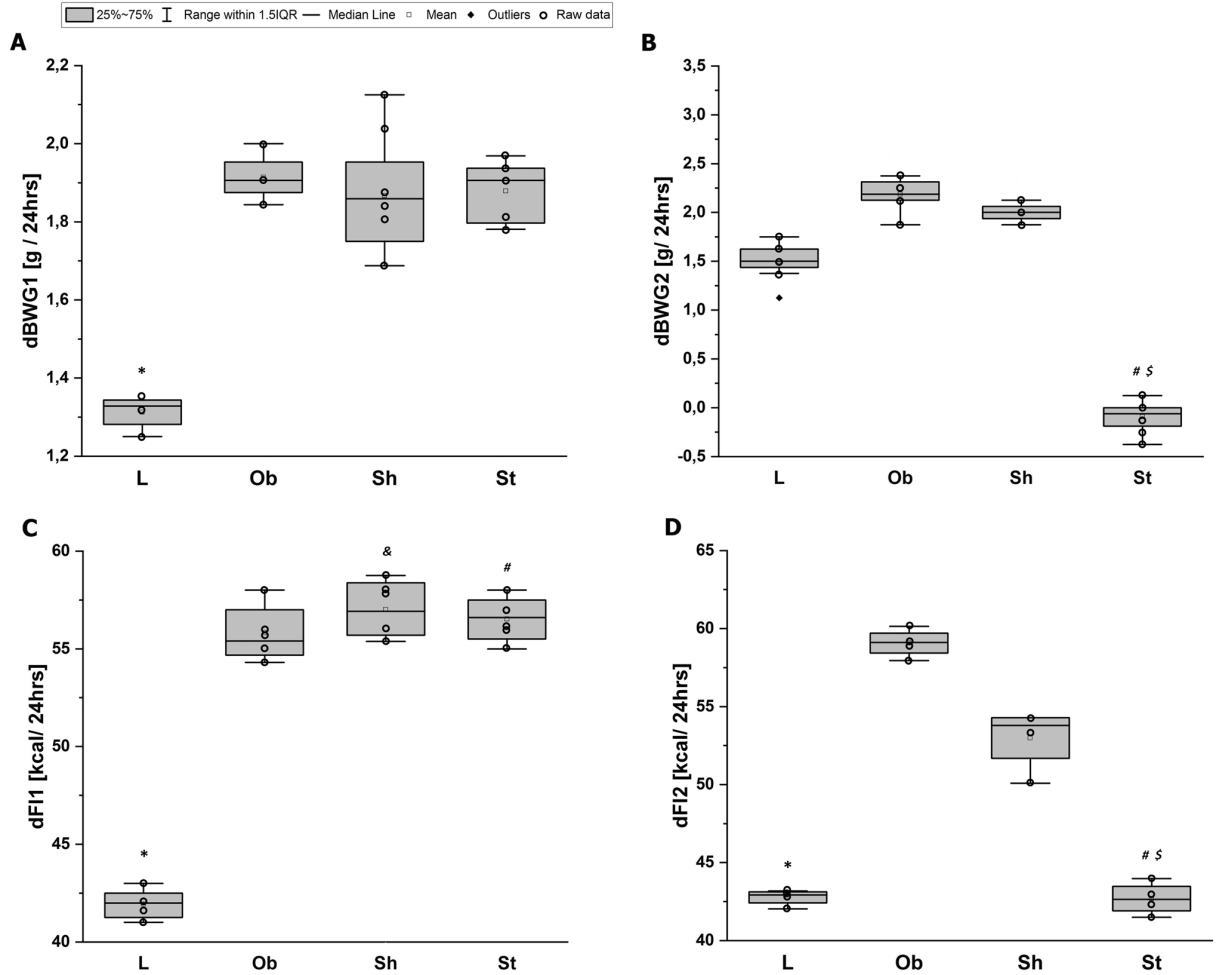
Average daily body weight gain within groups [g/24 h] at: **A** pre-stimulation period (dBWG1) and **B** stimulation period (dBWG2). Average daily feed intake within groups [kcal/24 h] during: **C** pre-stimulation period (dFI1), and **D** stimulation period (dFI2). L—lean intact (*N* = 8); Ob—intact with obesity (*N* = 8); Sh—sham-stimulated with obesity (*N* = 8); St—obese with active stimulation (*N* = 8). Kruskal–Wallis test followed by the Bonferroni post hoc test. Data are shown as median (25–75th percentile). *p < 0.007 vs. (Ob + Sh + St), ^$^*p* < 0.03 vs. Sh, ^**#**^*p* < 0.05 or ^&^*p* = 0.008 vs. (L + Ob)

**Fig. 4:**
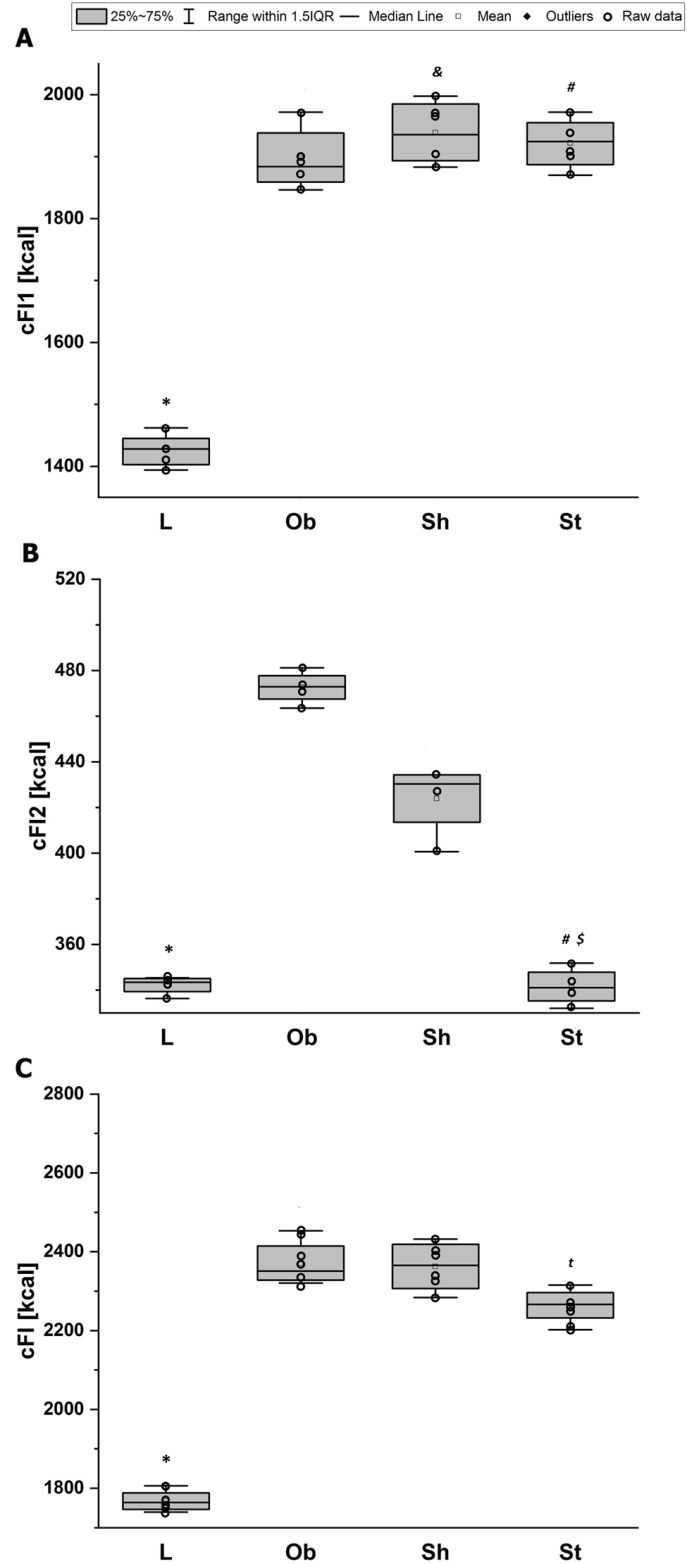
Cumulative feed intake [kcal] at: **A** pre-stimulation period (cFI1), **B** stimulation period (cFI2), **C** the whole study (cFI): L—lean intact (*N* = 8); Ob—intact with obesity (*N* = 8); Sh—sham-stimulated with obesity (*N* = 8); St—obese with active stimulation (*N* = 8). Kruskal–Wallis test followed by the Bonferroni post hoc test. Data are shown as median (25–75th percentile). *p < 0.01 vs. (Ob + Sh + St), ^**#**^*p* < 0.05 or ^&^*p* = 0.008 vs. (L + Ob), ^t^*p* = 0.065 or ^$^*p* = 0.023 vs. Sh

**Fig. 5:**
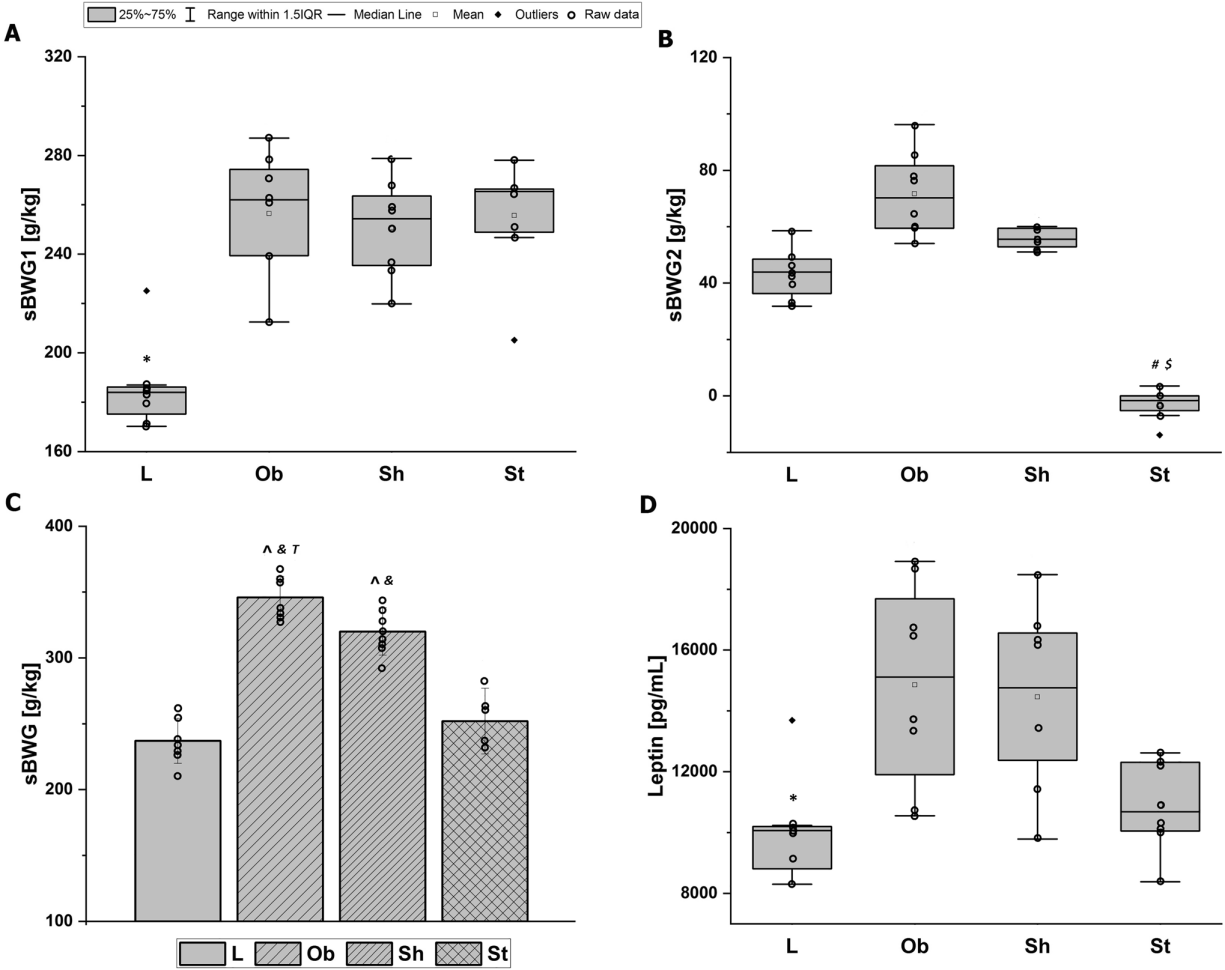
Specific rate of body weight gain [g/kg] at: **A** pre-stimulation period (sBWG1), **B** stimulation period (sBWG2), **C** the whole study (sBWG) as well as **D** serum leptin concentrations [pg/mL] at the end of the study. L—lean intact (*N* = 8); Ob—intact with obesity (*N* = 8) Sh—sham-stimulated with obesity (*N* = 8); St—obese with active stimulation (*N* = 8). Two-way ANOVA (sBWG) or Kruskal–Wallis test (sBWG1, sBWG2, leptin) followed by the Bonferroni post hoc test. Data are shown as mean ± SD (sBWG) or median (25–75th percentile). ^*p* = 0.000 vs. L, ^&^*p* = 0.000 vs. St, *p < 0.006 vs. (Ob + Sh + St), ^**#**^*p* = 0.000 vs (L + Ob), ^$^*p* = 0.001 or ^T^*p* = 0.059 vs. Sh

The average initial body weight did not differ among the tested groups: *F*_3,28_ = 0.3, *p* = 0.77 (Fig. 2A). As expected, throughout almost 5 weeks of pre-stimulation period, the L rats consumed fewer calories than the animals on a specific diet with a higher fat content as the average daily feed intake (dFI1, kcal/24 h) and consequently cumulative feed intake (cFI1, kcal) significantly differed between the groups: *H* = 18, *N*_1_ = 8, *N*_2_ = 24, *p* = 0.000 and *H* = 18, *N*_1_ = 8, *N*_2_ = 24, *p* = 0.000, respectively (Figs. 3C, 4A). Increased calorie consumption (dFI1) went along with higher daily body weight gain (dBWG1, g/24 h) (correlation coefficient *r*_S_ = 0.40, *N* = 32, *p* = 0.023), indicating overeating as dominant cause of obesity in our rats. The body weight and feed intake did not differ among subgroups on HCD at the pre-stimulation period (Figs. 2A, 3A, C). Application of active AtDCS for 8 consecutive days changed both feed intake and body weight resulting in reduced daily (dFI2, kcal/24 h) (*H* = 9, *N*_1_ = 16, *N*_2_ = 8, *N*_3_ = 8, *p* = 0.013) and therefore cumulative calorie consumption (cFI2, kcal) (*H* = 9, *N*_1_ = 16, *N*_2_ = 8, *N*_3_ = 8, *p* = 0.011) as well as daily body weight gain (dBWG2, g/24 h) (*H* = 18, *N*_1_ = 16, *N*_2_ = 8, *N*_3_ = 8, *p* = 0.000) (Figs. 3B, D, 4B). A post hoc pairwise comparison using the Bonferroni correction revealed a significant difference in dFI2, cFI2 and dBWG2 between St and intact animals (L and Ob): *p* = 0.029 and *p* = 0.024 and *p* = 0.000, respectively, as well as when comparing St with sham stimulation (Sh): *p* = 0.024 and *p* = 0.023 and *p* = 0.001, respectively. Specific rate of BWG (sBWG, g/kg) calculated in the groups additionally underlined the impact of both HCD and active AtDCS on body weight: *F*_3,28_ = 60, *p* = 0.000 (Fig. 5A–C). No interaction between diet and stimulation procedure (“DIET” x “AtDCS”) on body weights was revealed, underlying their independent influence on measured variables (BW0, BW1, BW2, sBWG).

A repeated-measures ANOVA determined that mean body weight (BW, g) differed significantly across three time points: at the beginning of the study (BW0), just before the first stimulation (BW1) and at the end of the experiment (after the last stimulation) (BW2): (*F*_2,62_ = 918, *p* = 0.000) (Fig. 2A). A post hoc pairwise comparison using the Bonferroni correction showed an increased BW between the initial assessment (BW0) and the first (BW1) (232 vs 287 g, respectively) or the second (BW2) (232 vs 299 g, respectively) follow-up assessment as well as between the first (BW1) and the second (BW2) follow-up assessment (287 vs 299 g, respectively) (Fig. 2A). All of them reached significance (*p* = 0.000). Therefore, we can conclude that the results for the ANOVA indicate a strongly significant time effect for BW at both pre- and stimulation period. In addition, a significant strong interaction could be reported between TIME and GROUP: *F*_6,6_ = 56; *p* = 0.000 (Fig. 2A).

**Fig. 6:**
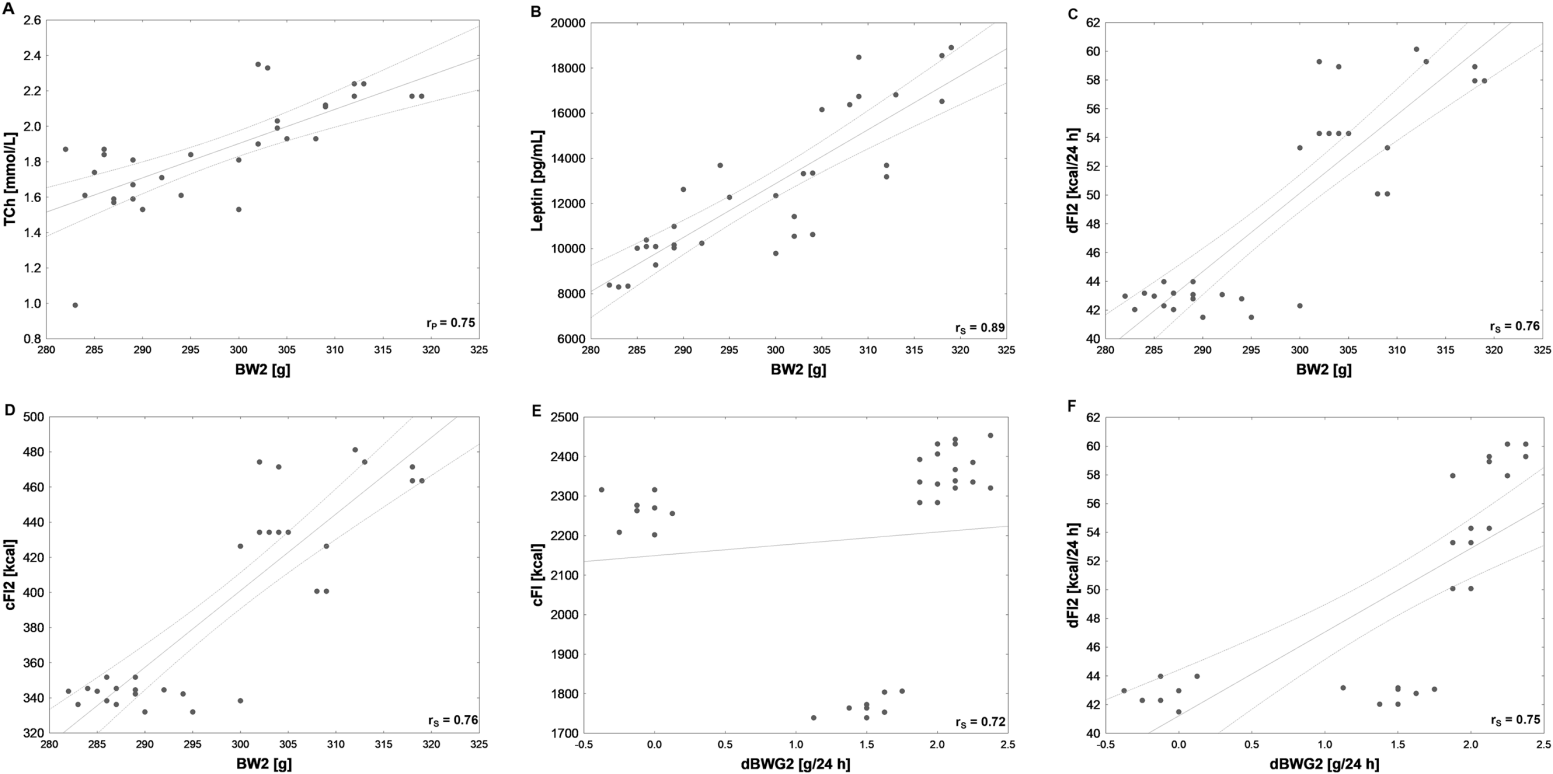
High (*r* > 0.7, *N* = 32) Pearson’s (*r*_P_) or Spearman’s (*r*_S_) correlations between body weight at the end of the experiment (BW2) and: **A** serum total cholesterol (TCh) and **B** leptin concentrations, **C** daily feed intake at stimulation period (dFI2), **D** cumulative feed intake at stimulation period (cFI2), as well as between daily body weight gain at stimulation period (dBWG2) and: **E** cumulative feed intake at the whole experiment (cFI) and **F** daily feed intake at stimulation period (dFI2). Both TCh and leptin concentrations were measured at the end of the study

Consumption of HCD increased serum leptin (pg/mL) (*H* = 8, *N*_1_ = 8, *N*_2_ = 24, *p* = 0.005) (Fig. 5D), TCh (mmol/L) (*F*_3,28_ = 20, *p* = 0.000) and LDL (mmol/L) levels (*H* = 17, *N*_1_ = 8, *N*_2_ = 24, *p* = 0.000) (Fig. 2B), but did not change HDL (mmol/L) or Tg (mmol/L) measured at the end of the study. Serum leptin concentration did not differ depending on applied stimulating procedure, although the Spearman’s correlation demonstrated very high positive (*r*_S_ = 0.89; *N *= 32, *p* = 0.000) relationship between serum leptin levels and body weights of the animals at the end of the study (Fig. 6B). Interestingly, AtDCS ameliorated serum lipoproteins resulting in significantly decreased TCh (*F*_3,28_ = 20, *p* = 0.000) and HDL (*H* = 7, *N*_1_ = 16, *N*_2_ = 8, *N*_3_ = 8, *p* = 0.03) with no influence on LDL or Tg (Fig. 2B). A post hoc pairwise comparison using the Bonferroni correction showed a significant difference in TCh between St and Sh (*p* = 0.003) and between intact animals (L and Ob) and Sh (*p* = 0.012). HDL levels significantly differed between St and Sh (*p* = 0.048) and had a tendency to be lower in St compared to intact animals (L and Ob) (*p* = 0.067). Sham stimulation had no impact on serum HDL levels. By analyzing the possible interaction between diet and stimulation procedure (“DIET” x “AtDCS”) on TCh, two-way ANOVA showed that the influence of individual variables is additive, it means independent of each other.

**Fig. 7:**
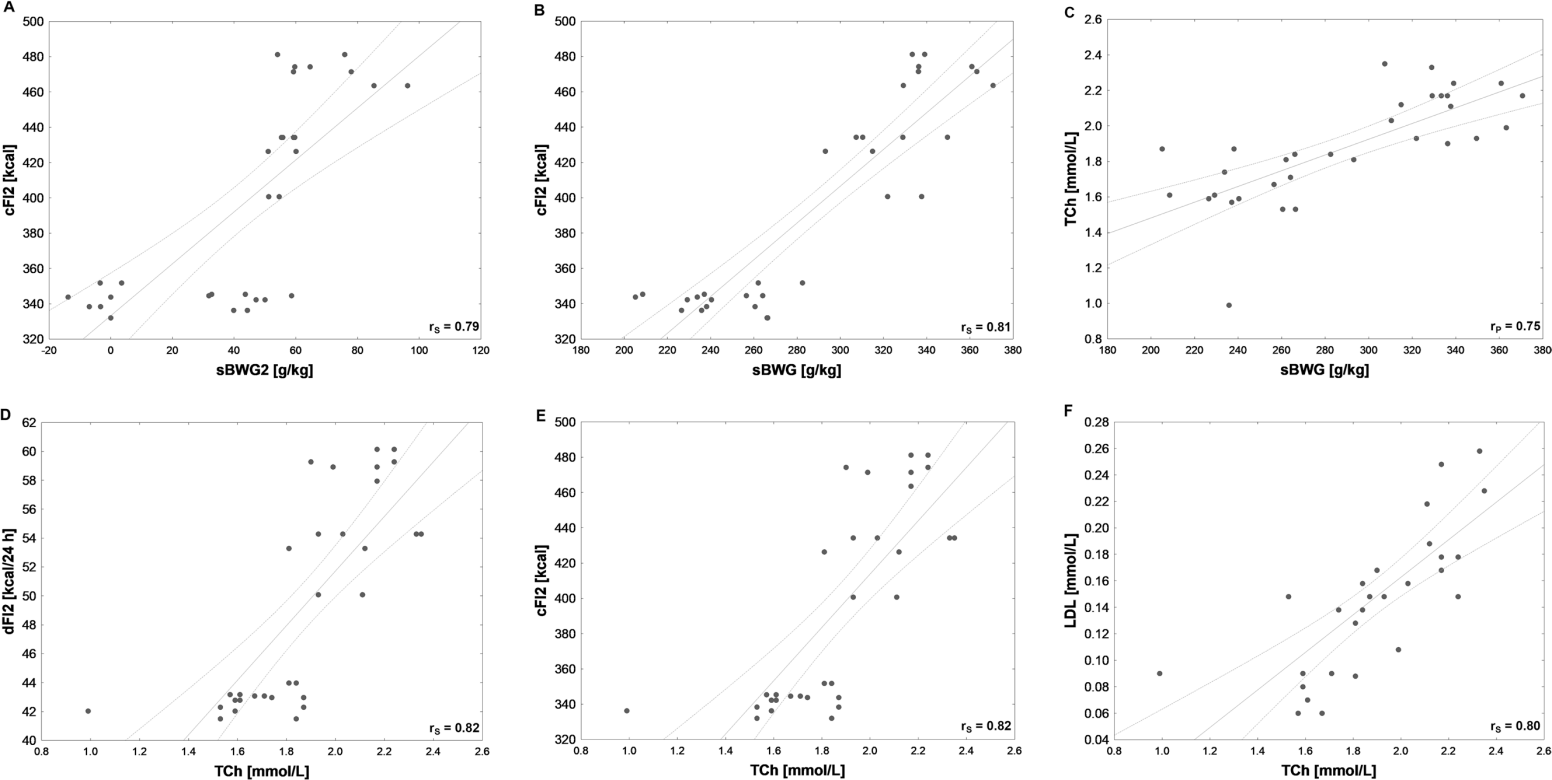
High (*r* > 0.7, *N* = 32) Pearson’s (*r*_P_) or Spearman’s (*r*_S_) correlations between: **A** specific rate of body weight gain at stimulation period (sBWG2) and cumulative feed intake at stimulation period (cFI2), **B** specific rate of body weight gain at the whole experiment (sBWG) and cumulative feed intake at stimulation period (cFI2), **C** specific rate of body weight gain at the whole experiment (sBWG) and concentration of serum total cholesterol (TCh), **D** concentration of serum total cholesterol (TCh) and daily feed intake at stimulation period (dFI2), **E** concentration of serum total cholesterol (TCh) and cumulative feed intake at stimulation period (cFI2), **F** concentration of serum total cholesterol (TCh) and concentrations of low-density lipoprotein (LDL). Both TCh and LDL concentrations were measured at the end of the study

**Fig. 8:**
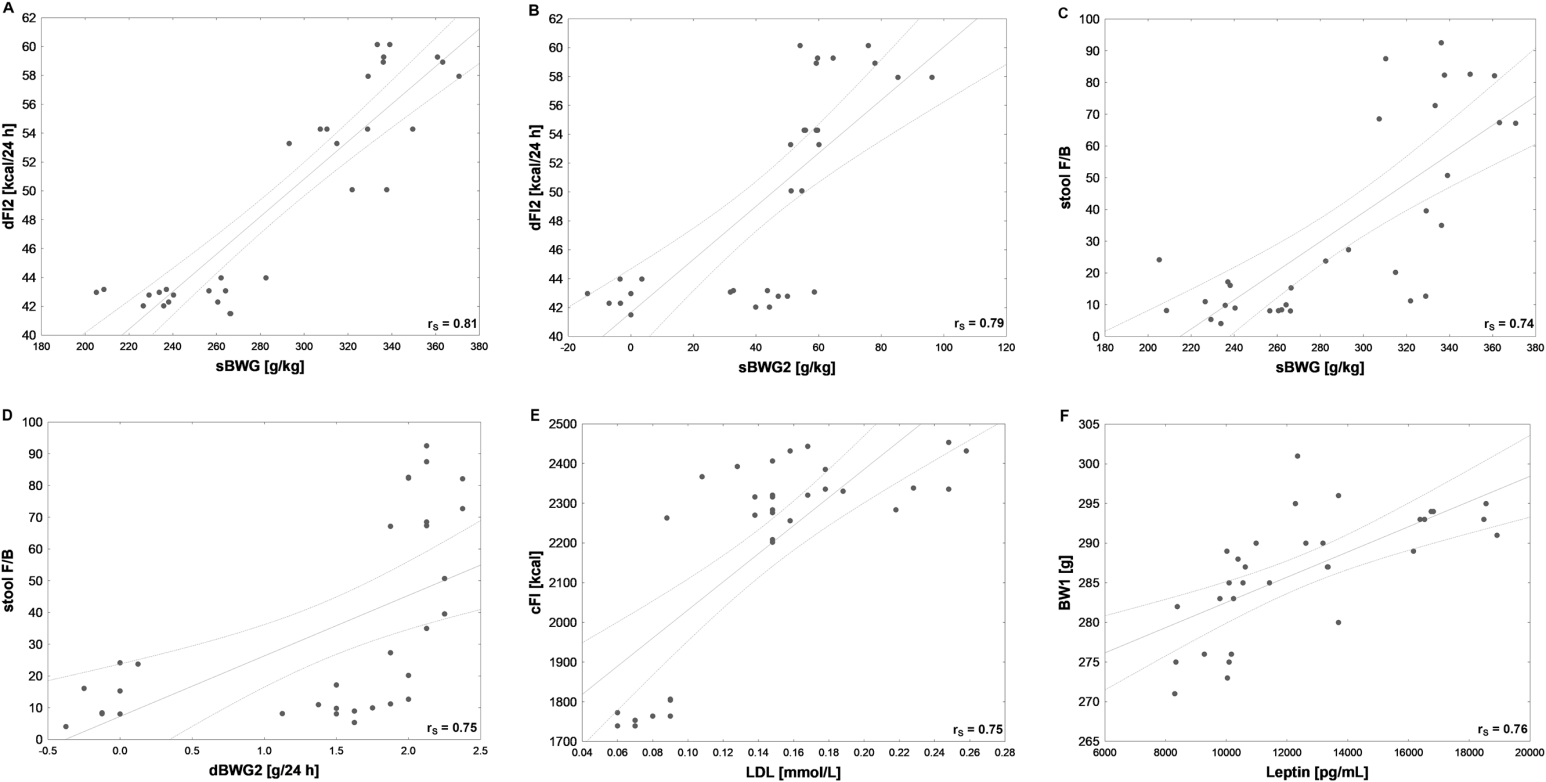
High (*r* > 0.7, *N* = 32) Pearson’s (*r*_P_) or Spearman’s (*r*_S_) correlations between: **A** specific rate of body weight gain at the whole experiment (sBWG) and daily feed intake at stimulation period (dFI2), **B** specific rate of body weight gain at stimulation period (sBWG2) and daily feed intake at stimulation period (dFI2), **C** specific rate of body weight gain at the whole experiment (sBWG) and the ratio of averaged numbers of Firmicutes to Bacteroidetes in stool (stool F/B ratio), **D** daily body weight gain at stimulation period (dBWG2) and the ratio of average number of Firmicutes to average number of Bacteroidetes in stool (stool F/B ratio), **E** concentrations of leptin (leptin) at the end of the study and body weight just before the first stimulation (BW1)

Several strong correlations between behavioral and metabolic parameters were detected in our tested groups (Figs. 6, 7, 8). BW2 was very highly positively correlated with dFI2 and therefore cFI2, as well as with serum leptin and TCh (Fig. 6). Very high positive relationships between dBWG2 and dFI2 or cFI2 were detected (Fig. 6). In addition, both sBWG and sBWG2 were very highly positively correlated with dFI2 and therefore cFI2 (Figs. 7, 8), while sBWG was related to TCh, as well (Fig. 6). Additionally, dFI2 and, therefore, cFI2 highly positively corresponded to TCh (Fig. 7). A very high relationships between TCh and LDL and between LDL and cFI2 are illustrated in Figs. 7 and 8.

### The assessment of selected phyla of gut microbiota

**Fig. 9:**
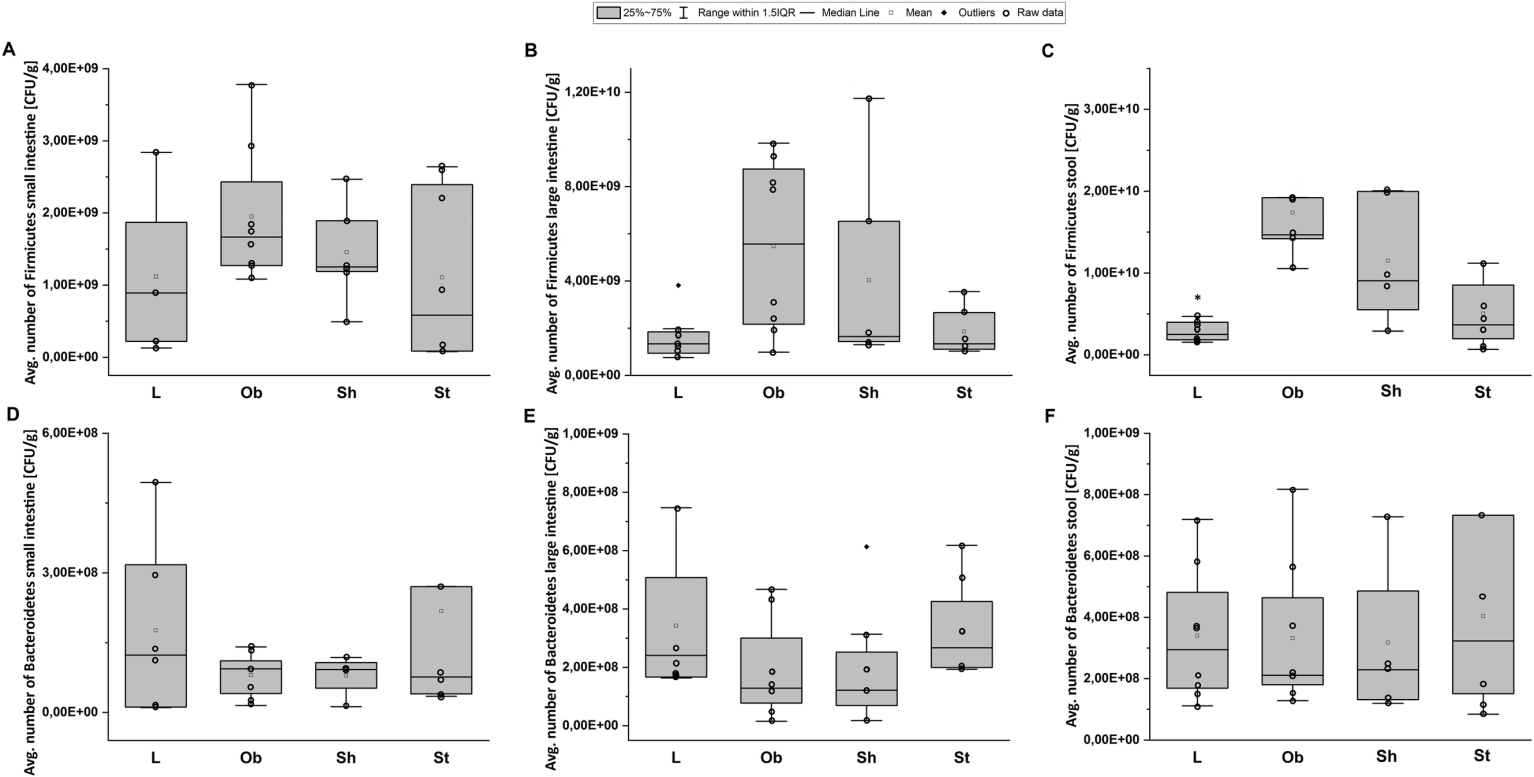
Average number of Firmicutes [CFU/g] and average number of Bacteroidetes [CFU/g] in small intestine (**A** and **D**, respectively); average number of Firmicutes [CFU/g] and average number of Bacteroidetes [CFU/g] in large intestine (**B** and **E**, respectively) and average number of Firmicutes [CFU/g] and average number of Bacteroidetes [CFU/g] in stool (**C** and **F**, respectively): L—lean intact (*N* = 8); Ob—intact with obesity (*N* = 8); Sh—sham-stimulated with obesity (*N* = 8); St—obese with active stimulation (*N* = 8); CFU—Colony Forming Unit. Kruskal–Wallis test followed by the Bonferroni post hoc test. Data are shown as median (25–75th percentiles). *p = 0.006 vs. (Ob + Sh + St)

**Fig. 10:**
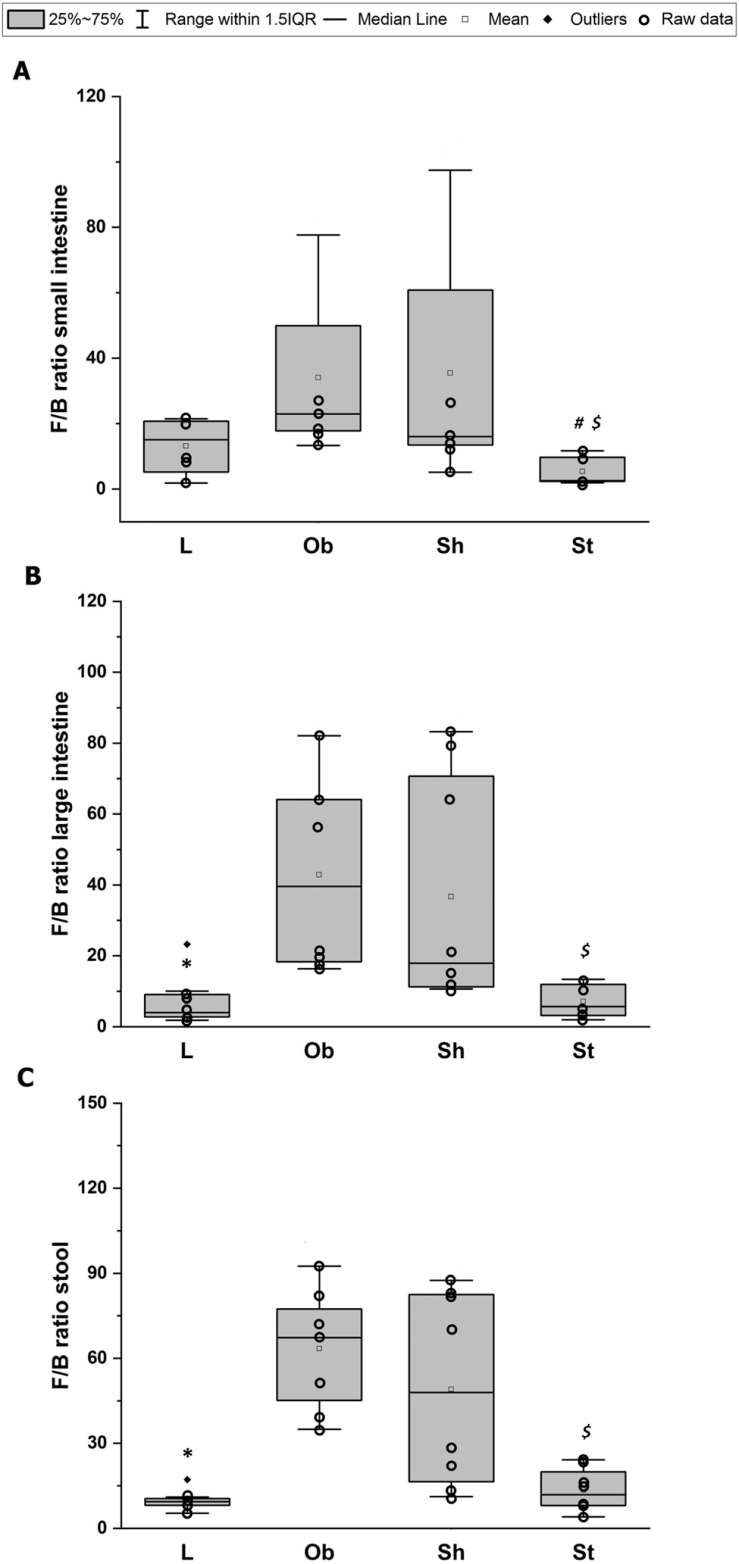
Ratio of average number of Firmicutes to average number of Bacteroidetes (F/B ratio) in small (**A**) and large (**B**) intestine as well as in stool (**C**). L—lean intact (*N* = 8); Ob—intact with obesity (*N* = 8); Sh—sham-stimulated with obesity (*N* = 8); St—obese with active stimulation (*N* = 8). Kruskal–Wallis test followed by the Bonferroni post hoc test. Data are shown as median (25–75th percentile). *p < 0.02 vs. (Ob + Sh + St); ^#^*p* = 0.007 vs. (L + Ob), ^$^*p* < 0.04 vs. Sh

The most prominent changes in microbiota composition secondary to HCD application were observed in the large intestine and stool of our rats. High-fat diet ingestion resulted in increased amounts of Firmicutes in the stool (*H* = 7, *N*_1_ = 8, *N*_2_ = 24, *p* = 0.006) (Fig. 9C) leading to a greater stool F/B ratio (*H* = 8, *N*_1_ = 8, *N*_2_ = 24, *p* = 0.004) (Fig. 10C). HCD also increased an F/B ratio in large intestine significantly (*H* = 7, *N*_1_ = 8, *N*_2_ = 24, *p* = 0.011) (Fig. 10B).

Active AtDCS of the right PFC changed gut and stool main phyla quantity resulting in reduced F/B ratios in small intestine (*H* = 11, *N*_1_ = 8, *N*_2_ = 24, *p* = 0.004), large intestine (*H* = 7, *N*_1_ = 8, *N*_2_ = 24, *p* = 0.033) and stool (*H* = 6, *N*_1_ = 8, *N*_2_ = 24, *p* = 0.039) (Fig. 10A–C). A post hoc pairwise comparison using the Bonferroni correction showed a significant difference in a small intestine F/B ratio between St and Sh (*p* = 0.012) or between St and intact animals (L and Ob) (*p* = 0.007), in a large intestine F/B ratio between St and Sh (*p* = 0.029) as well as in a stool F/B ratio between St and Sh (*p* = 0.033). No significant differences were detected in microbiota composition between intact (L and Ob) and sham-stimulated animals in all examined tissues (Fig. 9). Spearman’s correlation revealed a very high relationship between a stool F/B ratio and sBWG (*r*_s_ = 0.74, *N* = 32, *p* = 0.000) (Fig. 8C) or dBWG2 (*r*_s_ = 0.75, *N* = 32, *p* = 0.002) (Fig. 8D).

## Discussion

The new finding of our study is that repeated AtDCS of the right PFC is effective not only in ameliorating obesity-related feeding behavior and metabolic disturbances, but also gut microbiota composition in rats on high-calorie diet. These outcomes prove the existence of sophisticated network of nervous, metabolic, immune and other connections between brain and intestines known as brain–gut–microbiome axis. To our knowledge, this is the first study evaluating microbiome composition in tDCS-treated versus control subjects with obesity.

Obesity is growing health and financial problem all over the word [56]. It has been shown that widespread available the so-called Western diet rich in simple sugars and saturated fats as well as sedentary lifestyle are currently the main causes of excessive body adiposity. Due to unsatisfactory effectiveness of pharmacological treatment and invasiveness of bariatric operation new safe and efficient strategies for obesity treatment are searched for. tDCS is a non-invasive method to deliver weak currents to the brain through two surface electrodes (anode and cathode) for altering cortical excitability which is believed to affect deeper brain regions [57]. tDCS seems to provoke a shift in membrane potentials of stimulated cortical regions for minutes to hours depending on the duration and polarity of tDCS [58, 59]. Anodal tDCS increases facilitation after the stimulation session, not during it. Cathodal tDCS, in turn, attenuates facilitation during the stimulation period and increases inhibition after it. [60].

Stimulation protocol applied in the current experiment was proved to be safe and effective in our previous studies [50, 51] which did not reveal any macroscopic or microscopic evidence of brain abnormalities. Moreover, animals subjected to the stimulation showed no behavioral anomalies or signs of discomfort. In addition, parameters of tDCS (current intensity, duration and number of stimulation cycles, diameters and location of active and counter electrodes) were shown not to be harmful [60–62]⁠. Several studies pointed at the right hemisphere as a critical area involved in appetite control [37, 38]. Damage to the right frontal lobe can result in hyperphagia and a specific preference for fine food [63] while hyperactivity of this brain area, like in patients with epilepsy, can lead to anorexia-like symptoms [64]. In addition, in patients with degenerative dementia, the presence of hyperphagia correlates positively with right frontal atrophy [65] and obese individuals show a reduced right-hemispheric preference in motor and cognitive tasks [66]. On the other hand, some data have shown that also left or bilateral [67–69] PFC stimulation resulted in decreased food cravings in women. We have recently shown that both anodal right and cathodal left tDCS of the prefrontal cortex are able to modify feeding behavior in male obese Wistar rats [50, 51, 69].

In the present study, we have shown that both HCD and AtDCS can affect feeding behavior, metabolic parameters as well as main phyla of the gut microbiota of female Wistar rats. Because sex and species differences in the development of diet-induced obesity and metabolic disturbances in rodents were described [70], we aimed at investigating if our feeding and stimulation protocols [50, 51, 69] were effective regardless of gender. It was shown [70] that diet-induced hyperphagia is greater in males offered a high-fat diet, irrespective of species, and female rats show a delay in diet-induced obesity and metabolic complications due to higher energy expenditure and lower level of hyperphagia. Furthermore, obese female and male rodents obtained a dramatic adiposity and glucose intolerance, probably due to a decreased energy expenditure and to higher intake of saturated fats compared to the lean controls. The results are consistent with our observations as in the present study female rats consumed less calories (56 vs 76 kcal/24 h, respectively) and gained their body weight slower (1.9 vs 2.8 g/24 h, respectively) than their male counterparts from our previous study [50]. Despite lower end body weight of female rats, serum leptin concentrations were comparable in both sexes indicating that our female rodents had relatively more leptin and perhaps more fat tissue. Serum TCh, Tg and LDL concentrations were comparable in our male and female rats while HDL seemed to be lower (2.0 vs 1.6 mmol/L, respectively) and additionally ameliorated by AtDCS in females.

Five weeks of HCD in pre-stimulation period significantly increased daily and therefore cumulative feed intake resulting in increased body weight gain in the rats on specific diet compared with their lean counterparts. These outcomes point at increased caloric consumption as a dominant cause of obesity in our rats and are consistent with generally accepted view that obesity results from increased energy supply [71]. The average feed intake did not differ before and during stimulation period inside all the groups except St indicating AtDCS of the right PFC as an effective tool to restrict appetite in obese rats. Indeed, only rats with active AtDCS changed their feeding behavior consuming less calories than other rats during stimulation period and also when comparing the pre-stimulation (dFI1) and stimulation time (dFI2) (56.5 ± 1.2 kcal/24 h vs 42.7 ± 1.0 kcal/24 h, respectively). Additionally, AtDCS-induced caloric intake inhibition went along with decreased rate of body weight gain pointing at reduced feed consumption as a dominant cause of decreased body weight in our rats. Our results are in line with data showing that AtDCS of the right PFC is able to restrict food cravings in both animals [56] and humans [72]. Additionally, it was shown that even one session of the dorsolateral frontal cortex tDCS [45, 46] is effective in a significant reduction in food craving. As it was expected [73] HCD application increased serum leptin levels which highly positively correlated with end body weights (BW2) (Fig. 6). Our results prove the commonly observed findings that serum leptin levels positively correlate with BMI [74] and body adiposity in humans and animals [75–77] as white adipose tissue is the main source of leptin. The arcuate nucleus (Arc) of the hypothalamus is a key region involved in regulation of food intake and responding to circulated leptin [78]. One important target for leptin action is Arc CART/POMC (cocaine- and amphetamine-regulated transcript/proopiomelanocortin) neurons which expression is inhibited in ob/ob mice and fasted rodents compared to the controls and this is reversed. Arc CART/POMC mRNA expression is normalized by leptin administration [79] suggesting that these leptin-sensitive neurons may be critical in regulating leptin-induced thermogenesis and energy expenditure. In contrast to POMC/CART, Arc AGRP (agouti-related peptide) and NPY (neuropeptide Y) mRNAs are negatively regulated by leptin [80]. Central administration of both NPY and AGRP increases feeding and this increase is prevented by leptin administration showing the presence of leptin receptors on the NPY/AGRP cells [81]. In these mechanisms, leptin inhibits food intake and increases energy expenditure. However, most cases of human obesity are characterized by resistance to leptin action therefore no benefits from hyperleptinemia are observed.

HCD and obesity attenuated lipid profile in our rats, as TCh and LDL increased compared with control levels. Interestingly, AtDCS application ameliorated TCh and HDL but not LDL or Tg concentrations resulting in significantly reduced both TCh and HDL levels compared with both sham- stimulated and intact rats. These observations indicate that alterations in serum HDL did not depend on a diet but rather stimulation procedure. Contrary to HDL, TCh and LDL, positively correlated with end body weight (BW2), body weight gain (sBWG), daily (dFI) and cumulative feed intake (cFI) as well as leptin levels pointing to the causative relationship between obesity-related behavioral and metabolic parameters.

Finally, our results revealed that not only HCD but also AtDCS application influenced selected phyla of gut microbiota and the changes were associated with alternations in behavioral and metabolic parameters. Recent investigations suggest that an altered composition and diversity of gut microbiota, due to impairment in energy homeostasis, could play an important role in the development of neuropsychiatric [82] and metabolic disorders, such as obesity [83, 84] Gut microbiota play a number of physiological roles involving digestion, metabolism, extraction of nutrients, synthesis of vitamins, prevention against colonization by pathogens, and immunomodulation [25, 85]. The main bacterial phyla are: firmicutes (gram positive), bacteroidetes (gram negative), and actinobacteria (gram positive). Gut microbiota composition may be transiently altered by diet, disease, drugs, stress and environment [85].

Our diet rich in fat change the amount of Bacteroidetes and Firmicutes in large intestine and stool resulting in a significantly elevated F/B ratio in the examined tissues. Our results are consistent with numerous studies which have demonstrated the relationship between obesity or an increased fat intake and an increase in the Gram-positive/Gram-negative index of gut microbiota [86, 87]. In Ob/Ob mice with a mutation in the leptin gene, the proportion of the dominant gut phyla, Bacteroidetes and Firmicutes, is modified with a significant reduction in Bacteroidetes and a corresponding increase in Firmicutes [87]. The shift in the relative abundance observed in these phyla is associated with the increased capacity to harvest energy from food and with increased low-grade inflammation. In another study [22], the Western diet increased the relative abundance of Firmicutes at the expense of the Bacteroidetes and the changes in microbiome composition were totally reversed after a shift back to  a standard diet indicating that altered gut microbiota composition is rather a cause and not a consequence of obesity or altered dietary habits [22]. Data from human studies are generally consistent with the results from animal models showing that obese subjects had lower Bacteroidetes and more Firmicutes in their large intestine than lean control humans did [84]. The F/B ratio was noted significantly higher in obese subjects, with a value more than twice that in normal-weight patients [88]. In addition, Bacteroidetes abundance is increasing along with weight loss of people with obesity [83], whereas Firmicutes phylum, especially some of their genera as Lactobacillus and Clostridium, have been associated with obesity-related metabolic dysregulations [89]. The application of AtDCS to our obese rats affected the amounts of examined phyla in the tested tissues. Although AtDCS have changed an average number of Bacteroidetes and Firmicutes irrelevantly, it resulted in a significantly reduced F/B ratio compared with sham stimulation. This observation suggests that AtDCS is able to reverse the adverse effects of HCD and obesity on the gut microbiome. AtDCS-induced improvement of the main microbiota phyla toward control levels points to this non-invasive technique as an effective tool for modulation of gut microbiota contents in obese subjects. Moreover, the findings support the hypothesis of the existence of complex bidirectional communication between the brain and intestine known as the brain–gut–microbiome axis [90, 91]. Several pathways, including the vagus nerve, the immune and endocrine system, as well as the enteric nervous system, mediate the bidirectional communication between the brain and the gut microbiota [92]. The vagus nerve, which is a major afferent pathway from the abdominal cavity to the CNS, seems to be the fastest and most direct way for the microbiota to influence the brain [92, 93]. There are reports suggesting that the microbiome may activate this pathway, influencing brain action at the physiological and behavioral level [94]. One of the possible explanation of HCD or AtDCS effects on feeding behavior, metabolic parameters and selected microbiome phyla in our study could be the contribution of the vagus nerve as a signal transductor between the brain and gut, as the 10th cranial nerve is known to be involved in hunger and satiety regulation [72, 94]. Mucosal vagal afferents respond to a variety of chemical stimuli from gastrointestinal tract and gut microbiota can alter vagus nerve signaling by influencing the releasing of these factors from gastrointestinal cells or producing neurotransmitters such as serotonin which is involved in appetite control [95]. tDCS was shown to modulate cravings for food by influencing some brain neurotransmitters releasing [82]. We have recently demonstrated [50] that both serotonin and dopamine are involved in tDCS-induced feed restriction in obese rodents. Brain also can affect microbiota composition and function by alteration of the mucus layer where bacteria grow by modulating gut motility, secretion of acid and bicarbonates, intestinal fluid handling and mucosal immune response, as well as intestinal permeability, allowing bacterial antigens to penetrate the epithelium and stimulate an immune response in the mucosa [92]. AtDCS-induced improvement of the main microbiome phyla in our obese rats to control levels suggests a potential role of AtDCS in restoring the luminal habitat/normal mucous and therefore the integrity of tight junctions protecting the intestinal barrier.

Numerous relationships were identified between selective phyla of the gut microbiome and some physiological parameters. Interestingly, the highest positive correlation was identified between a stool F/B ratio and body weight gain (both dBWG2 and sBWG) pointing at this ratio as one of the appropriate marker of obesity. To confirm our observations, further research should be extended to the analysis of species belonging to the Bacteroidetes and Firmicutes phyla with the use of the new generation sequencing method.

Some of the measured effects of AtDCS on feeding behavior, metabolic parameters and selected phyla contents in the gut were slightly blunted by sham stimulation suggesting the possible impact of stress associated with applied procedure on achieved results. However, most of the end-points of the study were significantly changed by the active AtDCS pointing at this technique as a possible therapeutic tool of obesity.

## Conclusions

We concluded that AtDCS of the right PFC is capable to modulate feeding behavior, metabolic parameters and gut microbiome composition in obese rats on HCD. These findings prove the existence of the bidirectional communication between the brain and intestines known as the brain–gut–microbiome axis and underline the role of microbiota composition in the pathogenesis of obesity. In addition, our results point to the tDCS as a promising and non-invasive complementary treatment of obesity, although the obtained behavioral and metabolic effects, as well as the optimal stimulation protocol of AtDCS application, should be further investigated and replicated in human studies.

## Abbreviations

AGRP: Agouti-related peptide
Arc: Arcuate nucleus of the hypothalamus
AtDCS: Anodal transcranial direct current stimulation
Apo B: Apolipoprotein B
BMI: Body mass index (kg/m^2^)
BW: Body weight (g)
BW0: Initial body weight (g) at the beginning of the experiment
BW1: Body weight (g) just before the first stimulation
BW2: Body weight (g) at the end of the experiment (after the last stimulation)
CART: Cocaine- and amphetamine-regulated transcript
cFI: Cumulative feed intake (kcal) during the whole experiment
cFI1: Cumulative feed intake (kcal) during pre-stimulation period
cFI2: Cumulative feed intake (kcal) during stimulation period
CtDCS: Cathodal transcranial direct current stimulation
CFU: Colony forming units
dBWG: Daily body weight gain (g/24 h) during the whole experiment
dBWG1: Daily body weight gain (g/24 h) during pre-stimulation period
dBWG2: Daily body weight gain (g/24 h) during stimulation period
dFI: Daily feed intake (kcal/24 h) during the whole experiment
dFI1: Daily feed intake (kcal/24 h) during pre-stimulation period
dFI2: Daily feed intake (kcal/24 h) during stimulation period
DLFC: Dorsolateral frontal cortex
F/B ratio: Firmicutes to Bacteroidetes ratio
HCD: High-calorie diet
HDL: Low-density lipoprotein (mmol/L)
LDL: Low-density lipoprotein (mmol/L)
NPY: Neuropeptide Y
PFC: Right prefrontal cortex
POMC: Proopiomelanocortin
qPCR: Quantitative PCR real-time amplification
sBWG: Specific rate of body weight gain (g/kg) during the whole experiment
sBWG1: Specific rate of body weight gain (g/kg) during pre-stimulation period
sBWG2: Specific rate of body weight gain (g/kg) during stimulation period
TCh: Total cholesterol (mmol/L)
Tg: Triglycerides (mmol/L)
VLDL: Very low-density lipoproteins
WAT: White adipose tissue

## Declarations

### Data availability

The data that support the findings of this study will be made available from the corresponding author after reasonable request up to 5 years after publication. We may require the participation in the authorship after the use of the shared data.

### Code availability

No applicable.
